# Measuring clinical outcomes in children with pediatric acute-onset neuropsychiatric syndrome: data from a 2–5 year follow-up study

**DOI:** 10.1186/s12888-021-03450-5

**Published:** 2021-10-04

**Authors:** Caroline De Visscher, Eva Hesselmark, Daniel Rautio, Ida Gebel Djupedal, Maria Silverberg, Selma Idring Nordström, Eva Serlachius, David Mataix-Cols

**Affiliations:** 1 Centre for Psychiatry Research, Department of Clinical Neuroscience, Karolinska Institutet, Stockholm, Sweden; 2 Stockholm Health Care Services, Region Stockholm, CAP Research Center, Gävlegatan 22, SE-113 30, Stockholm, Sweden; 3 Moment Psykologi, Drottninggatan 99, 113 60 Stockholm, Sweden

**Keywords:** PANS, PANDAS, OCD, Tourette, Immunopsychiatry

## Abstract

**Background:**

It is unclear how to best measure the complex symptom presentation of pediatric acute-onset neuropsychiatric syndrome (PANS).

**Methods:**

Well-characterized participants of a 2–5 year follow-up study (*n* = 34; 56% male) underwent clinical evaluations and completed scales assessing global symptom severity, functional impairment and specific psychiatric symptoms. We explored inter-correlations between the measures and used intraclass correlation coefficients to evaluate the agreement between clinician-, parent- and child ratings of the same constructs.

**Results:**

Ratings on symptom-specific measures varied largely between participants. Agreement between informants was excellent on functional scales, fair-to-moderate on global severity scales and mixed on symptom-specific scales. Clinician-rated global and functional measures had stronger inter-correlations with parent- and child-rated functional measures than with symptom-specific measures.

**Conclusions:**

General instruments assessing global severity and functioning are well suited for the assessment and follow-up of PANS, but should be complemented by symptom-specific scales representative of core symptoms.

**Supplementary Information:**

The online version contains supplementary material available at 10.1186/s12888-021-03450-5.

## Introduction

Pediatric acute-onset neuropsychiatric syndrome (PANS) is a descriptive entity consisting of acute-onset OCD and/or eating disorder accompanied by a wide range of secondary psychiatric and somatic symptoms [1]. Initial and recurring symptoms may be severe and lead to significant loss of function [2, 3]. There are no clearly established evidence-based treatments for PANS [4] but the long-term prognosis is generally positive, with approximately two thirds of patients presenting with minimal or no symptoms 2–5 years after initial presentation [5]. However, approximately one third of patients have a chronic clinical course and require additional treatment [5].

Several measures have been proposed for the characterization of PANS patients [6–8]. The wide range of symptoms represented within the PANS construct constitutes a challenge for both daily clinic work and for the design of clinical trials [4]. At least two clinician-rated instruments or symptom checklists have been specifically developed for PANS, but their administration is time-consuming, their items cannot be easily collated to calculate total scores, and their psychometric properties have not been established [9, 10]. The use of gold standard measures for specific psychiatric symptoms, such as the Children’s Yale-Brown Obsessive Compulsive Scale (CY-BOCS) [11] or the Yale Global Tic Severity Scale (YGTSS) [12, 13] as single outcome measures is also problematic because they only capture specific symptom clusters and their use may result in both an over- or underestimation of treatment responders [4, 14]. Conversely, trying to assess every individual symptom cluster may result in an overwhelming number of rating scales for the families to complete, reducing the quality of the collected data and the willingness to participate in research.

Previous experience from our PANS cohort [3, 5] suggested a potential discrepancy between clinician-rated measures of global functioning, disease severity and improvement scales on the one hand, and subjective reports from parents on the other. It is possible that frequently used instruments may not capture the full extent of the patients’ difficulties [5]. For some families this may result in frustration and a perceived lack of understanding of the true impact of PANS from the medical community.

In this study, we aimed to investigate the suitability of standard clinical measures for the assessment and follow-up of children with PANS, and formally assess the degree of agreement between multiple informants (child, parent and clinician ratings). Ultimately, we aim to shed some light on the optimal ways to measure the complex presentation of this patient group.

## Methods

### Participants

Participants were consecutive referrals to a multidisciplinary immunopsychiatry outpatient clinic in Stockholm, Sweden, who were previously included in a Swedish PANS cohort [3, 5]. Cohort members who had a minimum of 2 years since inclusion were eligible for participation in a follow-up study, regardless of whether they were still active patients in the clinic or not. The current analyses are based on data collected as part of the follow-up study, i.e. 2–5 years after first assessment [5]. All participants met strict PANS criteria at first assessment.

Parents/guardians of the participants gave written informed consent to participate in the study, which was approved by the Regional Ethics Review Board in Stockholm (reference number EPN 2015/1977–31/4 (2019–02132)). All procedures performed in the study were in accordance with the 1964 Declaration of Helsinki and its later amendments.

### Clinical evaluations

A child and adolescent psychiatrist conducted a 2-h face-to-face assessment, including a standardized patient- and parent interview and clinician-rated measures of symptoms and general functioning. Child- and parent-rated measures of specific symptoms and general functioning were completed prior to the visit to the clinic. Some of the participating children were very young, but parents were instructed to help their children as little as possible when compiling their responses. In the few cases where an item on a scale was missing, these were completed via a telephone call with the parent. If a child-rated item was missing, the parent was instructed to report the missing item in collaboration with the child.

The specific measures were chosen based on our clinical experience and recommendations from the 2013 PANS Consensus Conference [7], and can be classified as measures of global symptom severity and adaptive functioning, and symptom-specific measures.

### Measures of global symptom severity and adaptive functioning

The *Clinical Global Impressions-Severity scale (CGI-S)* is a clinician-rated scale measuring the current severity of a patients psychiatric illness in general, on a 7 point single-item scale [15]. CGI-S scores range from *‘normal’* (score 1) to *‘extremely ill’* (score 7). It is a widely used, validated clinical outcome measure in psychiatry [16]. In addition to the original clinician-rated version, the CGI-S was adapted for its use as a self-report measure, resulting in parent- and child-rated versions of the scale (see Supplemental material).

The *Strengths and Difficulties Questionnaire – Parent−/Self-rated version (SDQ-P/S)* is a validated parent- and child-rated, behavioral screening questionnaire consisting of five subscales, four measuring difficulties (hyperactivity, emotional symptoms, conduct problems and peer problems) and one measuring strengths (pro-social behavior). Items are scored from *‘not true’* (score 0) to *‘certainly true’* (score 2), with a maximum total difficulty score of 40. A higher total difficulty score indicates a higher symptom burden and a lower global functioning. A total difficulty score of 14 has been suggested as a cut-off. SDQ-S is adapted for 11–16 year old children and adolescents and was therefore only used for this age group in the study [17, 18].

The *Children’s Global Assessment Scale (CGAS)* is a clinician-rated, extensively validated, single-item measure of general functioning. The scale ranges from 1 to 100, with higher scores indicating a higher (better) level of general functioning [19]. The assessment should be made by a specifically trained child and adolescent psychiatrist or psychologist after a thorough clinical assessment and reflect the most impaired level during a specified time period of 1 month, regardless of treatment and/or prognosis [20, 21].

*KIDSCREEN-10 – Parent/Youth version* is a parent- and child-rated screening instrument measuring well-being and health-related quality of life for children and adolescents. The scale contains 10 items, using a 5-point response scale, with a maximum score of 50. A higher score indicates higher (better) health-related quality of life. An additional item measuring general well-being is scored separately, using a 5-point scale, from *‘bad’* (score 1) to *‘excellent’* (score 5) [22, 23].

The *Work and Social Adjustment scale- Parent/Youth version (WSAS-P/Y)* is a brief and reliable parent- and child-rated measure of educational, work and social adjustment in children and adolescents. The scale includes five items related to everyday activities (school and employment, everyday activities, social activities, leisure time, and family/relationships), scored from *‘not impaired at all’* (score 0) to *‘severely impaired’* (score 8), with a maximum score of 40. WSAS-P/Y has high internal consistency and is sensitive to change [24, 25].

### Symptom-specific measures

The *Children’s Yale-Brown Obsessive Compulsive Scale (CY-BOCS)* and the *Yale Global Tic Severity Scale (YGTSS)* are clinician-rated instruments to quantify the severity of OCD and tic disorder symptoms, respectively [11–13]. Both scales have excellent psychometric properties and are routinely employed in clinical practice and clinical trials. Higher total scores indicate a higher symptom burden, and clinically significant levels of OCD and tic symptoms are generally considered to be reached at CY-BOCS > 15 and YGTSS > 30 [12, 26, 27].

The *Obsessive Compulsive Inventory-Child Version (OCI-CV)* is a 21-item child-rated self-report measure of OCD symptom severity that correlates moderately well with clinician-rated measures of OCD symptoms. It consists of seven sub-scales (doubting/checking, obsessing, hoarding, washing, ordering and neutralizing). Items are scored from ‘never’ (score 0) to ‘always’ (score 2), with a maximum score of 42. A higher score indicates more severe symptoms [28, 29].

The *Short Moods and Feelings Questionnaire – Parent/Child version (SMFQ-P/C)* is a 13-item parent- and child-rated screening tool for depression in children and adolescents, developed from the longer 34-item version Moods and Feelings Questionnaire (MFQ). Both versions of the scale have been extensively validated in community and clinical samples. Responses are rated using a 3-point scale from *‘not true’* (score 0) to *‘true’* (score 2), with a maximum score of 26. A higher score indicates more severe depressive symptoms. Suggested cut-offs for girls are > 16 and for boys > 5 when parent- or self-rated [30, 31].

The *Separation Anxiety Avoidance Inventory – Parent/Child version (SAAI-P/C)* is a validated 12-item parent- and child-rated measure of avoidance behavior in separation situations. Each item is scored from *‘never’* (score 0) to *‘always’* (score 4), with a maximum score of 48. A higher total score indicates more severe avoidance behavior [32].

The *Insomnia Severity Index – Child and adolescent version (ISI-C)* is a 7-item child-rated measure assessing insomnia severity using a 5-point scale from *‘not at all’* (score 0) to *‘extremely’* (score 4), with a maximum score of 28. A high total score indicates greater insomnia severity, with scores 15 and above indicating clinical insomnia [33, 34].

The *Autism Spectrum Quotient Child/Adolescent version-10 (AQ-10)* is a 10-item parent-rated instrument initially developed as a tool to aid referral decision making for autism spectrum disorder (ASD) evaluation. The maximum score is 10 and scores 6 or above are considered a positive indication of ASD [35]. Previous data has shown a high incidence of neuropsychiatric symptoms in PANS-patients during follow-up, highlighting the importance of using an ASD screening tool even for previously assessed patients [5].

The Swanson, Nolan and Pelham scale (SNAP-IV) is a parent-rated scale assessing attention deficit and hyperactivity (ADHD)-related symptoms and oppositional defiant disorder (ODD) [36, 37]. It is a frequently used tool in treatment studies as well as in daily clinic work following-up ADHD treatment. The SNAP-IV exists in different versions depending on items rated. The version most frequently used in Sweden is a 30-item version rating ADHD inattention, ADHD hyperactivity/impulsivity and ODD. Each item is scored from *‘not at all’* (score 0) to *‘very much’* (score 4), with a maximum total score of 120. Mean scores for each sub-section of the scale are also calculated [38].

The *Eyberg Child Behavior Inventory (ECBI)* is a 36-item parent-rated scale of disruptive behavior problems in children, divided in an intensity scale measuring the frequency of a behavior and a problem scale measuring if the parent perceives the behavior as a problem. The maximum score on the intensity scale is 252, and scores > 130 are considered clinically significant. The maximum score on the problem scale is 36, with scores > 14 indicating significant parental distress [39, 40].

### S***tatistical analysis***

For each scale and subscale, we calculated descriptive statistics (median, mean and SD). Intraclass correlation coefficients (ICCs) were calculated to establish the degree of agreement between clinician-, parent- and child ratings of the same rating scale. Because the set of raters was different for each target (i.e. same clinician but different set of parent and child rating each patient), ICC (1,*k*) estimates and their 95% confident intervals were calculated based on a mean-rating by *k* number of raters (*k* = 3 if clinician, parent and child; *k* = 2 if clinician and parent, clinician and child or parent and child), absolute-agreement, one-way random effects model [41]. Individual ICCs (the agreement between different raters on the same individual/participant) are reported. ICC values < 0.40 correspond to poor agreement, values between 0.40 and 0.59 to fair agreement, values between 0.60 and 0.74 to good agreement, and values > 0.75 to excellent agreement [42]. Correlation coefficients were calculated in order to measure the degree of association between measures. Because the data were not always normally distributed, Spearman rank correlation was used. Correlation coefficients between 0 and 0.29 represent poor association, 0.30–0.49 fair association, 0.50–0.79 moderate association and > 0.80 a very strong association [43, 44].

All statistical analyses were conducted using STATA software (version STATA/IC15.1 for Mac, StataCorp LLC, Texas, USA). *P*-values below 0.05 were considered to be statistically significant.

## Results

### Sample characteristics

Thirty-four out of 46 eligible PANS patients consented to participate in the follow-up study and provided data. Median age at follow-up was 11.5 years (range 6.7–17.1) and 19 (56%) of the participants were male. Further details on the clinical characteristics of the cohort, including duration of illness, comorbidities, current symptoms, family history of psychiatric and autoimmune disease can be found in a previous publication [5].

### Descriptive statistics

At a group level, median and mean scores for most global and specific symptom scales generally indicated low-to-moderate symptom severity and high level of functioning. However, there was substantial variability in the data, suggesting that some individuals experienced impaired symptoms at follow-up. Descriptive statistics for each of the measures are presented in Tables 1 and 2.

**Table 1:** Descriptive statistics of global severity and adaptive function rating scales at follow-up (*n* = 34)

	n	median (range)	mean (SD)
CGI-S^a^ clinician	34	3 (1–6)	2.8 (1.4)
CGI-S parent	34	2 (0–5)	2.4 (1.5)
CGI-S child	33	1 (0–6)	2.2 (1.6)
SDQ-P^b^ total	34	9.5 (3–23)	11.1 (6.4)
SDQ-P emotional symptoms	34	3.5 (0–10)	4 (2.3)
SDQ-P hyperactivity/inattention	34	4.5 (0–10)	4.1 (3.1)
SDQ-P peer problems	34	1 (0–6)	1.6 (1.7)
SDQ-P conduct problems	34	1 (0–6)	1.4 (1.6)
SDQ-P prosocial behavior	34	9 (4–10)	8.2 (1.8)
SDQ-S^c^ total	22	10 (4–18)	10.3 (4.5)
SDQ-S emotional symptoms	22	4 (0–10)	3.8 (2.3)
SDQ-S hyperactivity/inattention	22	3.5 (0–8)	3.9 (2.2)
SDQ-S peer problems	22	1.5 (0–6)	1.5 (1.4)
SDQ-S conduct problems	22	1 (0–5)	1.3 (1.2)
SDQ-S prosocial behavior	22	9 (6–10)	8.7 (1.2)
CGAS^d^	34	61 (28–80)	60.9 (13.5)
KIDSCREEN-10 parent total	34	38.5 (25–47)	37.5 (5.8)
KIDSCREEN-10 parent general well-being	34	3 (1–5)	3.1 (1.1)
KIDSCREEN-10 child total	33	39 (24–46)	38.3 (6.1)
KIDSCREEN-10 child general well-being	33	3 (1–5)	3.3 (1)
WSAS-P^e^	34	12.5 (0–30)	12.9 (9.2)
WSAS-Y^f^	33	9 (0–31)	10.8 (9.7)

^a^*CGI-S* Clinical Global Impression-Severity scale

^b^*SDQ-P* Strengths and Difficulties Questionnaire-Parent rated

^c^*SDQ-S* Strengths and Difficulties Questionnaire-Self rated

^d^*CGAS* Children’s Global Assessment Scale

^e^*WSAS-P* Work and Social Adjustment Scale-Parent version

^f^*WSAS-Y* Work and Social Adjustment Scale-Youth version

**Table 2:** Descriptive statistics of symptom-specific rating scales at follow-up (*n* = 34)

	n	%	median (range)	mean (SD)
CY-BOCS^a^	34		8 (0–30)	8.2 (8)
YGTSS^b^	34		4.5 (0–65)	10.4 (14)
OCI-CV^c^	33		11 (0–18)	9.5 (5.1)
SMFQ-P^d^	34		4.5 (0–14)	5.9 (4.4)
SMFQ-C^e^	31		4 (1–19)	5.5 (4.7)
SAAI-P^f^	33		10 (0–35)	12.5 (10.9)
SAAI-C^g^	33		8 (0–36)	10.6 (9.4)
ISI-C^h^	33		5 (0–18)	5.9 (4.6)
AQ-10^i^	31		3 (0–8)	3 (2)
SNAP-IV^j^	34		18.5 (0–57)	21.9 (14.3)
SNAP-IV inattention	34		1 (0–2.3)	1.1 (0.7)
SNAP-IV hypertactivity/impulsivity	34		0.4 (0–2.4)	0.7 (0.7)
SNAP-IV ODD	34		0.5 (0–2.1)	0.7 (0.6)
ECBI^k^ intensity scale	33		90 (41–169)	95.9 (34.5)
ECBI problem scale	34		7 (0–25)	7.9 (7.4)

^a^*CY-BOCS* Children’s Yale-Brown Obsessive Compulsive Scale

^b^*YGTSS* Yale Global Tic Severity Scale

^c^*OCI-CV* Obsessive Compulsive Inventory-Child Version

^d^*SMFQ-P* Short Moods and Feelings Questionnaire-Parent version

^e^*SMFQ-C* Short Moods and Feelings Questionnaire-Child version

^f^*SAAI-P* Separation Anxiety Avoidance Inventory-Parent version

^g^*SAAI-C* Separation Anxiety Avoidance Inventory-Child version

^h^*ISI-C* Insomnia Severity Index-Child and Adolescent version

^i^*AQ-10* Autism Spectrum Quotient Child/Adolescent version-10

^j^*SNAP-IV* Swanson, Nolan and Pelham scale-IV

^k^*ECBI* Eyberg Child Behavior Inventory

### Agreement between informants

The overall ICC (1,3) of CGI-S ratings made by clinician, parent and child was 0.57 (95% CI 0.37–0.74). The ICC (1,2) of clinician and parent CGI-S ratings was 0.69 (0.46–0.83), the ICC (1,2) of clinician and child CGI-S ratings was 0.47 (95% CI 0.17–0.70) and the ICC (1,2) of parent and child CGI-S ratings was 0.55 (95% CI 0.26–0.75). Thus, the overall agreement between CGI-S ratings made by clinician, parent and child was only fair.

Regarding the SDQ-P/S for participants > 11 years (*n* = 22), the ICC (1,2) of parent and child ratings was 0.64 (95% CI 0.31–0.83), representing a moderately good agreement.

The ICC (1,2) of parent and child ratings of the KIDSCREEN-10 was 0.81 (95% CI 0.65–0.90) and of parent and child ratings of the WSAS-P/Y 0.82 (95% CI 0.66–0.91), both representing excellent agreements*.*

The ICC (1,2) of SMFQ-P/C ratings made by parent and child was 0.46 (95% CI 0.14–0.70), only representing a fair agreement. In contrast, the ICC (1,2) of SAAI-P/C ratings was 0.88 (95% CI 0.77–0.94), representing an excellent agreement.

### Correlations between measures

Inter-correlations between clinician-rated CGAS and both parent-rated SDQ-P and child-rated SDQ-S were poor, including subscales. There was a fair association between clinician-rated CGI-S and parent-rated total SDQ (*ρ* = 0.448, *p* < 0.008), but not at a subscale level. The parent-rated functional scales KIDSCREEN-10 and WSAS-P had stronger inter-correlations than did the general symptom-severity scale SDQ-P with clinician-rated global symptom and functional scales. There was a moderate-to-strong association between parent-rated KIDSCREEN-10 and clinician-rated CGI-S (*ρ* = − 0.663, *p* < 0.001) and a very strong association between WSAS-P and clinician-rated CGI-S (*ρ* = 0.811, p < 0.001). The same was true for the child-rated versions of the functional scales. See Tables 3 and 4 (subscales available in Tables S1 and S2).

**Table 3:** Spearman correlations between clinician-rated global symptom and functional scales and parent-rated KIDSCREEN-10, WSAS-P and SDQ-P and child-rated functional scales KIDSCREEN-10, WSAS-Y and SDQ-S, *n* = 34

Spearman correlation, ρ	CGAS	CGI-S clinician	CGI-S parent	KIDSCREEN-10 parent	WSAS-P	SDQ-P
CGAS^a^	1					
CGI-S^b^ clinician	−0.911	1				
CGI-S parent	−0.555	0.664	1			
KIDSCREEN-10 parent	0.567	−0.663	− 0.308	1		
WSAS-P^c^	−0.669	0.811	0.660	−0.710	1	
SDQ-P^d^	−0.374	0.448	0.423	−0.564	0.528	1

^a^*CGAS* Children’s Global Assessment Scale

^b^*CGI-S* Clinical Global Impression – Severity scale

^c^*WSAS-P* Work and Social Adjustment Scale Parent version

^d^*SDQ-P* Strengths and Difficulties Questionnaire Parent-rated

**Table 4:** Spearman correlations between clinician-rated global symptom and functional scales and child-rated KIDSCREEN-10, WSAS-Y and SDQ-S, *n* = 22 (excludes participants < 11 years)

Spearman correlation, ρ	CGAS	CGI-S clinician	CGI-S child	KIDSCREEN-10 child	WSAS-Y	SDQ-S
CGAS^a^	1					
CGI-S^b^ clinician	−0.929	1				
CGI-S child	−0.511	0.473	1			
KIDSCREEN-10 child	0.627	−0.626	− 0.257	1		
WSAS-Y^c^	−0.570	0.640	0.315	−0.569	1	
SDQ-S^d^	−0.304	0.215	0.344	−0.195	0.196	1

^a^*CGAS* Children’s Global Assessment Scale

^b^*CGI-S* Clinical Global Impression – Severity scale

^c^*WSAS-Y* Work and Social Adjustment Scale Youth version

^d^*SDQ-S* Strengths and Difficulties Questionnaire Self- rated

Clinician-rated global symptom and functional scales had moderate associations with clinician-scored CY-BOCS but not to child-rated OCI-CV. There was only a fair association between CY-BOCS and OCI-CV (ρ = 0.468, *p* < 0.006). See Table S3.

There was a fair-to-moderate association between clinician-rated CGAS and CGI-S and both parent- and child-rated SMFQ-P/C. Symptoms of separation anxiety and sleep disorder were more uncommon in the cohort, and inter-correlations between the symptom-specific measures SAAI-C and ISI-C and the global symptom and functional scales were lower. See Table 5.

**Table 5:** Spearman correlations between clinician-rated global and functional scales and SMFQ-P/C, SAAI-P/C and ISI-C, *n* = 30

Spearman correlation, ρ	CGAS	CGI-S clinician	SMFQ-P	SMFQ-C	SAAI-P	SAAI-C	ISI-C
CGAS^a^	1						
CGI-S^b^ clinician	−0.904	1					
SMFQ-P^c^	−0.447	0.516	1				
SMFQ-C	−0.582	0.457	0.527	1			
SAAI-P^d^	−0.362	0.485	0.461	0.146	1		
SAAI-C	−0.241	0.364	0.258	0.871	0.125	1	
ISI-C^e^	−0.198	0.297	0.362	0.297	0.225	0.253	1

^a^*CGAS* Children’s Global Assessment Scale

^b^*CGI-S* Clinical Global Impression – Severity scale

^c^*SMFQ-P/C* Short Moods and Feelings Questionnaire-Parent/Child version

^d^*SAAI-P/C* Separation Anxiety Avoidance Inventory-Parent/Child version

^e^*ISI-C* Insomnia Severity Index Child/Adolescent version

There was a fair-to-moderate association between CGAS and CGI-S and AQ-10, ECBI and SNAP-IV, specifically on the SNAP-IV inattention subscale. ECBI had a very strong association to SNAP-IV, highest on the SNAP-IV total but also on hyperactivity and conduct subscales, as expected. See Table 6.

**Table 6:** Spearman correlations between clinician-rated global and functional scales and AQ-10, SNAP-IV and ECBI, *n* = 30

Spearman correlation, ρ	CGAS	CGI-S clinician	AQ-10	SNAP-IV	SNAP-IV inattention	SNAP-IV hyper	SNAP-IV conduct	ECBI	ECBI problem
CGAS^a^	1								
CGI-S^b^ clinician	−0.904	1							
AQ-10^c^	−0.421	0.558	1						
SNAP-IV^d^	−0.398	0.511	0.330	1					
SNAP-IV inattention	−0.463	0.562	0.289	0.813	1				
SNAP-IV hyper	−0.238	0.300	0.220	0.916	0.652	1			
SNAP-IV conduct	−0.261	0.300	0.240	0.760	0.391	0.668	1		
ECBI^e^	−0.331	0.432	0.378	0.874	0.660	0.809	0.745	1	
ECBI problem	−0.373	0.503	0.584	0.813	0.614	0.720	0.710	0.937	1

^a^*CGAS* Children’s Global Assessment Scale

^b^*CGI-S* Clinical Global Impression – Severity scale

^c^*AQ-10* Autism Spectrum Quotient Child/Adolescent version-10

^d^*SNAP-IV* Swanson, Nolan and Pelham scale-IV

^e^*ECBI* Eyberg Child Behavior Inventory

In summary, the global clinician-rated symptom and functional measures tended to have stronger inter-correlations with parent- and child-rated functional measures than with symptom-specific measures.

## Discussion

In this study we analyzed data from a PANS cohort that had been followed-up for 2–5 years after initial presentation [5]. We examined the correspondence between clinician, parent and child measures of global symptom severity, adaptive functioning and specific psychiatric symptoms. This is critical because it is still unclear how to best measure the complex symptom presentation of the syndrome. Using the appropriate outcome measures has important implications for both clinical practice and the design of clinical trials.

Overall, median ratings for measures assessing global symptom severity and adaptive functioning indicated low symptom burden and a rather high level of everyday functioning in our sample. However, there was a large variability in the data, particularly in the symptom-specific measures, reflecting the heterogeneity of symptom presentations and clinical courses that are characteristic of the syndrome. These findings confirm and extend the findings of our previous study on the same cohort [5]. Specifically, we had previously reported that approximately one third of participants in the follow-up study had clinically significant symptoms and required additional treatment.

CGI-S is a gold standard measure of psychiatric illness severity, most frequently assessed by the clinician. The modest agreement between CGI-S ratings across informants in our sample suggests that it may be helpful complementing the clinician rating with ratings made by the parent and child. Overall, agreement between ratings made by parent and child were excellent for functional scales, but only fair-to-moderate for global symptom severity and symptom-specific scales, with the exception of the separation anxiety measure, which had excellent agreement between informants.

As expected, because of the previously mentioned heterogeneity of symptom presentations, global clinician-rated symptom and functional measures tended to have stronger inter-correlations with parent- and child-rated functional measures than with symptom-specific ones.

Parent- and child-rated functional scales KIDSCREEN-10 and WSAS-P/Y correlated well with clinician-rated global symptom and functional scales, and the agreements between ratings made by parent and child were excellent. Despite both scales being useful in the study, KIDSCREEN-10 may be more easily accessible to a younger patient group. Multiple parents commented on the suitability of the WSAS-P/Y, and indicated that its items may be less suitable for the younger patients. By contrast, the KIDSCREEN-10 was perceived as simple and straightforward for both younger children and teenagers.

Somewhat surprisingly, SDQ-P/S seemed to have weak inter-correlations with global symptom and functional scales and the agreement between informants was only moderate. This may be due to SDQ-P/S being more symptom-oriented than the other global measures used in the study. Results suggest that it may be less clinically useful in this particular patient group, but it should also be noted that the SDQ impact supplement was not used and therefore is not included in our analyses.

Obsessive-compulsive symptoms are part of main PANS criteria but were surprisingly rare in our sample at follow-up. Assessing obsessive-compulsive symptoms with a self-rated scale as a complement to CY-BOCS may not add a lot of information within the PANS patient group at follow-up. At onset, OCD is generally a more pervasive part of the symptom presentation, and more time should be devoted to a comprehensive OCD assessment.

We did not include a measure specifically measuring eating disorder symptoms because, in our clinical experience, these tend to be OCD-related and without the defining features of a typical eating disorder such as fears of gaining weight (resembling avoidant/restrictive food avoidance disorder). For selected patients with eating difficulties, it may be useful to measure these symptoms in order to track their improvement.

The agreement between SMFQ-P/C ratings made by parent and child was only fair, indicating the importance of having both parent- and child ratings of depression and thus avoid underestimating these symptoms in PANS patients. Conversely, the agreement between SAAI-P/C ratings made by parent and patient was excellent, suggesting that either parent or child ratings may suffice for clinical purposes.

Previous longitudinal data have shown a high comorbidity with neuropsychiatric disorders, combined with intensification of related symptoms during PANS flares [5, 45]. We therefore recommend measures that can screen for, and assess the severity of, autistic behaviors, inattention, hyperactivity and conduct problems when following up PANS. In our sample ECBI had a very strong association to SNAP-IV, suggesting that the simpler SNAP-IV may sufficiently cover the patients’ oppositional behaviors for clinical purposes. When detecting potentially severe oppositional defiant behaviors, ECBI can be used as a complement.

Our study has some limitations. First, we analyzed data from a small sample of patients from a single clinic. Second, the age range was such that the results of the child-rated measures should be interpreted with caution; despite our efforts, it is possible that the younger children received help from their parents to fill in their questionnaires. Third, we were not able to calculate internal consistency of the scales included in this study (only total scores were available). We could thus not examine the psychometric properties of the scales in this particular sample. Future studies would benefit from conducting such psychometric analyses.

## Summary

Clinical experience and the results from our follow-up study suggest that it is important to include clinician-, parent- and child ratings in the assessment of PANS, as a single perspective is unlikely to capture the full complexity of the syndrome. Brief, general measures assessing global disease severity and adaptive functioning, are clinically helpful and should be used but should be complemented by symptom-specific scales representative of the core symptoms in PANS, such as OCD, anxiety, depression and behavioral problems. However, their exclusive use is problematic as the natural course of the syndrome is such that some patients may not have specific symptoms to rate at follow-up. Furthermore, it is important that the assessment is straightforward and as brief as possible. Based on our experience, we recommend the use of a core battery of clinician-, parent- and child-rated measures in both clinical practice and in clinical trials (Table 7**)**. The proposed core battery of measures will provide a broad evaluation of PANS-related symptoms, but can be complemented with further symptom-specific measures when needed.

**Table 7:** Proposed rating scale protocol

***Clinician-rated scales:***	
CGAS^a^	
CGI-S/I^b^	
CY-BOCS^c^	
YGTSS^d^	
***Parent-rated scales:***	
CGI-S	
KIDSCREEN-10	
SMFQ-P^e^	
SAAI-P^f^	
AQ-10^g^	
SNAP-IV^h^	
***Child-rated scales:***	
CGI-S	
KIDSCREEN-10	
SMFQ-C	
ISI-C^i^	

^a^*CGAS* Children’s Global Assessment Scale

^b^*CGI-S* Clinical Global Impression – Severity scale

^c^*CY-BOCS* Children’s Yale-Brown Obsessive Compulsive Scale

^d^*YGTSS* Yale Global Tic Severity Scale

^e^*SMFQ-P/C* Short Moods and Feelings Questionnaire-Parent/Child version

^f^*SAAI-P* Separation Anxiety Avoidance Inventory-Parent version

^g^*AQ-10* Autism Spectrum Quotient Child/Adolescent version-10

^h^*SNAP-IV* Swanson, Nolan and Pelham scale-IV

^i^*ISI-C* Insomnia Severity Index Child/Adolescent version

## Supplementary Information

**Additional file 1.**

**Additional file 2.**

**Additional file 3.**

**Additional file 4.**

**Additional file 5.**

## Data Availability

The data are pseudonymised according to national (Swedish) and EU legislation, and cannot be anonymised and published in an open repository. The data can be made available upon reasonable request to Principal Investigator Professor David Mataix-Cols (david.mataix.cols@ki.se) on a case by case basis according to the current legislation and ethical permits.
