# Supplementary material for: Measuring clinical outcomes in children with pediatric acute-onset neuropsychiatric syndrome: data from a 2–5 year follow-up study
Source: BMC Psychiatry. 2021 Oct 4;21:484. doi: 10.1186/s12888-021-03450-5 (PMC8488538; doi:10.1186/s12888-021-03450-5)
Supplement: Supplementary file 1 — Additional file 1. [file 12888_2021_3450_MOESM1_ESM.docx]

**Supplemental material. CGI-S child version, English.**

CGI-S Child version

*Severity of illness*

How sick do you feel at present?

1. Normal, not at all ill
2. Borderline mentally ill
3. Mildly ill
4. Moderately ill
5. Markedly ill
6. Severely ill
7. Among the most extremely ill patients
