# Supplementary material for: Measuring clinical outcomes in children with pediatric acute-onset neuropsychiatric syndrome: data from a 2–5 year follow-up study
Source: BMC Psychiatry. 2021 Oct 4;21:484. doi: 10.1186/s12888-021-03450-5 (PMC8488538; doi:10.1186/s12888-021-03450-5)
Supplement: Supplementary file 2 — Additional file 2. [file 12888_2021_3450_MOESM2_ESM.docx]

**Supplemental material. CGI-S parent version, English.**

CGI-S Parent version

*Severity of illness*

According to Your experience of your child and his/her illness, how sick is your child at present?

1. Normal, not at all ill
2. Borderline mentally ill
3. Mildly ill
4. Moderately ill
5. Markedly ill
6. Severely ill
7. Among the most extremely ill patients
