# Supplementary material for: Measuring clinical outcomes in children with pediatric acute-onset neuropsychiatric syndrome: data from a 2–5 year follow-up study
Source: BMC Psychiatry. 2021 Oct 4;21:484. doi: 10.1186/s12888-021-03450-5 (PMC8488538; doi:10.1186/s12888-021-03450-5)
Supplement: Supplementary file 3 — Additional file 3. [file 12888_2021_3450_MOESM3_ESM.docx]

**Supplemental Table 1.** Correlations between clinician-rated global symptom and functional scales and parent-rated SDQ-P subscales, n=34.

| Spearman correlation, ρ | CGAS | CGI-S clinician | SDQ-P | SDQ-P emotional symptoms | SDQ-P hyperactivity | SDQ-P peer problems | SDQ-P conduct problems | SDQ-P prosocial behavior |
| --- | --- | --- | --- | --- | --- | --- | --- | --- |
| CGAS^a^ | 1 |  |  |  |  |  |  |  |
| CGI-S^b^ clinician | -0.911 | 1 |  |  |  |  |  |  |
| SDQ-P^c^ | -0.374 | 0.448 | 1 |  |  |  |  |  |
| SDQ-P emotional symptoms | -0.321 | 0.359 | 0.770 | 1 |  |  |  |  |
| SDQ-P hyperactivity | -0.203 | 0.257 | 0.876 | 0.565 | 1 |  |  |  |
| SDQ-P peer problems | -0.206 | 0.253 | 0.571 | 0.267 | 0.319 | 1 |  |  |
| SDQ-P conduct problems | -0.277 | 0.378 | 0.584 | 0.358 | 0.485 | 0.150 | 1 |  |
| SDQ-P prosocial behavior | 0.355 | -0.483 | -0.400 | -0.198 | -0.323 | -0.171 | -0.694 | 1 |

^a^CGAS: Children’s Global Assessment Scale

^b^CGI-S: Clinical Global Impression – Severity scale

^c^SDQ-P: Strengths and Difficulties Questionnaire Parent-rated
