# Supplementary material for: Measuring clinical outcomes in children with pediatric acute-onset neuropsychiatric syndrome: data from a 2–5 year follow-up study
Source: BMC Psychiatry. 2021 Oct 4;21:484. doi: 10.1186/s12888-021-03450-5 (PMC8488538; doi:10.1186/s12888-021-03450-5)
Supplement: Supplementary file 4 — Additional file 4. [file 12888_2021_3450_MOESM4_ESM.docx]

**Supplemental Table 2.** Correlations between clinician-rated global symptom and functional scales and self-rated SDQ-S subscales, n=22 (excludes participants <11 years).

| Spearman correlation, ρ | CGAS | CGI-S clinician | SDQ-S | SDQ-S emotional symptoms | SDQ-S hyperactivity | SDQ-S peer problems | SDQ-S conduct problems | SDQ-S prosocial behavior | |
| --- | --- | --- | --- | --- | --- | --- | --- | --- | --- |
| CGAS^a^ | 1 |  |  |  |  |  |  |  |  |
| CGI-S^b^ clinician | -0.929 | 1 |  |  |  |  |  |  |  |
| SDQ-S^c^ | -0.304 | 0.215 | 1 |  |  |  |  |  |  |
| SDQ-S emotional symptoms | -0.201 | 0.108 | 0.667 | 1 |  |  |  |  |  |
| SDQ-S hyperactivity | -0.048 | -0.445 | 0.650 | 0.166 | 1 |  |  |  |  |
| SDQ-S peer problems | 0.092 | -0.074 | 0.579 | 0.282 | 0.087 | 1 |  |  |  |
| SDQ-S conduct problems | -0.324 | 0.340 | 0.507 | 0.096 | 0.030 | 0.513 | 1 |  |  |
| SDQ-S prosocial behavior | 0.336 | -0.438 | -0.245 | -0.117 | 0.099 | -0.169 | -0.642 | 1 |  |

^a^CGAS: Children’s Global Assessment Scale

^b^CGI-S: Clinical Global Impression – Severity scale

^c^SDQ-S: Strengths and Difficulties Questionnaire Self- rated
