# Supplementary material for: Measuring clinical outcomes in children with pediatric acute-onset neuropsychiatric syndrome: data from a 2–5 year follow-up study
Source: BMC Psychiatry. 2021 Oct 4;21:484. doi: 10.1186/s12888-021-03450-5 (PMC8488538; doi:10.1186/s12888-021-03450-5)
Supplement: Supplementary file 5 — Additional file 5. [file 12888_2021_3450_MOESM5_ESM.docx]

**Supplemental Table 3.** Correlations between clinician-rated global and functional scales and CY-BOCS and OCI-CV, n=33.

| Spearman correlation, ρ | CGAS | CGI-S clinician | CY-BOCS | OCI-CV |
| --- | --- | --- | --- | --- |
| CGAS^a^ | 1 |  |  |  |
| CGI-S^b^ clinician | -0.915 | 1 |  |  |
| CY-BOCS^c^ | -0.660 | 0.710 | 1 |  |
| OCI-CV^d^ | -0.155 | 0.177 | 0.468 | 1 |

^a^CGAS: Children’s Global Assessment Scale

^b^CGI-S: Clinical Global Impression – Severity scale

^c^CY-BOCS: Children’s Yale-Brown Obsessive Compulsive Scale

^d^OCI-CV: Obsessive Compulsive Inventory-Child Version
